# Design and Measured Assessment of a MOS-Only, Capacitorless, Miniature 64-Channel Headstage Circuit for High-Density Surface Electromyography

**DOI:** 10.3390/s26134181

**Published:** 2026-07-02

**Authors:** Simos Koutsoftidis, Georgios Gryparis, Maciej Zajaczkowski, Guang Yang, Konstantinos Glaros, Dario Farina, Emmanuel M. Drakakis

**Affiliations:** The Department of Bioengineering, Imperial College London, London SW7 2AZ, UK

**Keywords:** HD sEMG, electromyography, multi-channel acquisition, analogue front-end, MOS-capacitors, pseudo-resistors

## Abstract

Background: We present a miniature (30 × 34 mm) 64-channel data acquisition headstage optimized for high-density surface electromyography. Methods: The headstage is made up of a multi-channel ASIC analogue front-end utilizing only MOS transistors, fabricated in 350 nm CMOS technology (IC die dimensions 6.9 × 1.8 mm), combined with an off-the-shelf multi-channel current-input ADC (DDC264, Texas Instruments). The ASIC analogue front-end employs MOS-based capacitors for both processing and AC-coupling. Results: The combination of these two sub-circuits enables the simultaneous recording of 64 channels at a typical sampling rate of 4 KHz with a maximum analogue bandwidth of 0.5–1500 Hz and a resolution of 20-bits. Typical input-referred-noise, determined by the analogue front-end, is 3.5 μV_RMS_ for a surface EMG bandwidth of interest of 20–500 Hz. This two-chip solution results in a power consumption of 5 mW per channel. Analogue performance variability of the custom ASIC was characterized across a dataset of 960-channels (15 dies) from two fabrication runs. Conclusions: This work practically demonstrates the viability of using both a MOS-only analogue front-end and commercially available off-shelf high-performance back-end hardware already developed for medical imaging applications to record high-density surface biosignals. The aforementioned techniques can be employed to reduce the size and cost for systems or wearable devices; facilitating the translation of high-density bio-acquisition setups from the research environment to more affordable commercial products.

## 1. Introduction

High-density surface electromyography (HD-sEMG) is a technique for recording the electrical activity generated by muscle activation, acquired non-invasively from the skin surface using arrays of closely spaced electrodes. These signals are typically acquired in unipolar derivation, using a common reference electrode placed away from the array. Typical acquisition bandwidth of interest ranges between 10–30 and 500 Hz, depending on the targeted muscle group and electrode configuration [1]. Signal amplitude typically ranges from hundredths of microvolts up to a few millivolts [2]. The experimental noise floor is typically limited by the skin-electrode noise, which exhibits levels on the order of 1–5 μV_RMS_ within the desired bandwidth under well-controlled lab conditions [1,2,3]. Mains interference may also be present on top of the sEMG recording as it falls within the typical acquisition bandwidth. Differential amplification can be used to attenuate mains interference levels down to below the skin-electrode noise level. An amplifier Common-Mode-Rejection-Ratio (CMRR) value of over 90 dB was thus proposed as a means to eliminate mains interference in the order of Volts for mains-powered acquisition systems with long analogue cables [1]. Practically, if the length of analogue traces between electrode and amplifier can be shorted down to a few centimeters by bringing the AFE very close to the electrode connector the amplitude of common-mode mains interference can be significantly reduced down to several millivolts. Hence, CMRR values of over 60 dB would be sufficient to significantly attenuate mains interference down to levels comparable to the AFE IRN. Battery-powered operation can also further aid in reducing the amount of mains-interference coupled to the system.

The HD sEMG approach provides significantly larger amounts of temporal and spatial information relative to the classic bipolar single-channel surface electromyography setup [4]. When combined with post-processing techniques, such as blind source separation, it is possible to decode the EMG signals into the activity of the innervating motor neurons [5]. Examination of motor unit activity by HD sEMG may be utilized in the study and diagnosis of neuromuscular conditions [6,7] or in man-machine interfacing applications, such as for hand gesture recognition [8,9], prosthetic control [10,11], and human authentication [12,13].

However, wearable HD sEMG interfacing systems have thus far remained within the scope of research and are not yet widely transferable to commercial or medical products. This is mainly due to the complexity, cost and size of the specialized acquisition hardware required for multi-channel recordings. A large portion of the cost of HD sEMG recording systems stems from the number of analogue sub-circuits required to amplify, filter and sample tens to hundredths of EMG channels simultaneously. Chip-scale integration of multi-channel acquisition modules has been attempted by research and commercial ventures as efforts to both miniaturize and reduce the overall cost of the acquisition system. Academic attempts over the last 25 years have mostly focused on the miniaturization of front-end circuits for implantable electroencephalography/electrocardiography (EEG/ECG) systems, where low power and small form-factor are most critical [14,15]. Several, low-power designs have been introduced capable of sampling from tens to hundredths of channels. Capacitive closed-loop operational transconductance amplifiers (OTAs) are typically utilized to achieve the desired area scaling due to their simplicity [16,17]. Chopper stabilization may sometimes be employed to reduce the contribution of amplifier flicker noise [18,19], which sometimes overlaps with the EEG and ECG spectra. EMG acquisition-specific hardware has not been as widely reported; however, the bandwidth and amplitudes of interest of EMG and ECG/EEG signals do have significant overlap, with typical ECG/EEG bandwidth ranging in the 0.5–150 Hz and EMG in 10–500 Hz (high-pass filter cutoff ranges from 10–30 Hz depending on specific sEMG applications). As of writing, the reported design methodologies have resulted in only a single commercially-available integrated circuit capable of filtering and acquiring several dozen biosignal channels at an integrated circuit (IC) footprint of <100 mm^2^ [20]. Being a niche product used predominantly for research applications, the per-channel cost of this IC is approximately 2–3 times higher than that of other commercially-available ICs with lower-channel count but comparable performance [21]. Moreover, commercially-available ICs supporting simultaneous acquisition of over 32 channels with a comparable footprint and performance do exist at a significantly lower per-channel cost for medical imaging applications [22]. Such devices have analogue front-ends optimized for acquiring micro-currents and hence cannot be directly applied to biosignal acquisition from the surface of the skin (where voltages are typically recorded with high input impedance amplifiers), despite their converter stages being perfectly suitable for this application. To further substantiate the point regarding performance characteristics, direct use of such ICs as Analogue Front-Ends (AFEs) has been demonstrated for implantable biosignal acquisition applications where current is directly sensed from tissues [23].

This work aims to showcase two specific solutions that can be applied, either individually or in-tandem, to reduce the cost per unit area of a multi-channel biosignal acquisition unit, focusing on HD sEMG as the example application.

Firstly, a simple, low-cost, 64-channel, analogue front-end has been implemented and fully characterized. Unlike in the vast majority of already published works discussed above, all integrated filters have been realized using MOS-based pseudo-resistors and MOS-capacitors; including, crucially, the input AC-coupling capacitors. The work presented here relies on previous key simulated and measured results presented in [24] and in [25] respectively. In [24] it has been shown, by means of heavy simulation studies, that the adoption of MOS-capacitors for the realization of high-order (e.g., 8th), biomimetic, class-AB Externally-Linear-Internally-Non-linear (ELIN) logarithmic and hyperbolic-sine One-Zero-Gammatone-Filter cochlea-like transfer functions, both results in aggressive reduction of capacitor area and preserves the very good linearity performance of the topologies despite the application of extremely high signal modulation indices. The latter simulation fact is of importance with respect to the promise of MOS-capacitors given the demanding, unforgiving and non-linear by design internal character of the logarithmic and hyperbolic-sine topologies; were the MOS-capacitors not appropriately biased and linear, the output distortion levels of those topologies would be very high. As far as the use of MOS-capacitors for the realization AC-coupling capacitors is concerned, a thorough literature search reveals that only a handful of works have proposed exploiting MOS capacitors to this extent [26,27,28,29]; with only a sub-set of those works actually providing measured results. Interestingly, the work presented in [25] assessed effectively the potential of the use of MOS-capacitors as AC-coupling elements by means of measured results from a few dies that each comprised a low number of EMG channels. More specifically: the measured results in [25] confirmed the significant reduction in area thanks to the AC-coupling MOS-capacitors, the achievement of very good linearity levels and the ability of the test channels (which were AC-coupled by means of MOS-capacitors) to accommodate differential offset voltages of ±250 mV for input signal levels of 5 mVp-p. On the other hand, despite the very good simulated and measured resultsin [24,25], the assessment of the approach by means of measurements from multi-channel systems across many dies remains to be seen and the work presented here progresses the field by assessing and focusing on this. The AC-coupling capacitor is typically the most area offending single device when considering the CC OTA. For example, in the case of the 350 nm technology used in this work, the adoption of MOS capacitors results in area savings ranging from 71% to 82% when compared to MIM or PIP components, respectively. Total system area savings become more pronounced in analogue area-hungry multi-channel systems. However, as mentioned above, the performance variability of this approach has yet to be characterized in significant number of channels/dies to validate its applicability for high density biosignal acquisition. The presented here MOS-only 64-channel solution was implemented using the mature 4-metal 350 nm CMOS semiconductor technology, a choice which apart from considerably reducing the area needs for the AC-coupling circuit via means of MOS capacitor, further helps reduce fabrication cost by requiring a lower number of masks in production.

**Figure 1:**
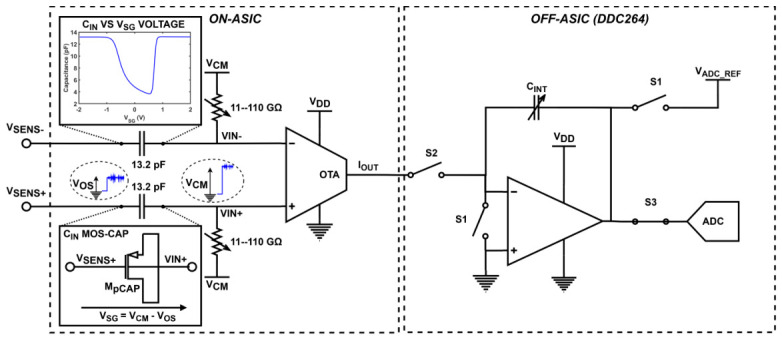
Proposed system-level design approach. The 13.2 pF input AC-coupling capacitors and 11–110 GΩ resistors are realized using pMOS devices (see Figure 2 and Table 1). The pMOS capacitors are biased at the common-mode voltage $V_{\mathrm{CM}}$ (1.5 V) to ensure a stable capacitance value for input offset voltages $V_{\mathrm{OS}}$ of up to hundredths of mV. Band-limited EMG signals are converted into nano-currents at $I_{\mathrm{OUT}}$ by the OTA block (Figure 3). These currents are sampled off-chip using a specialized current-input ADC (DDC264, Texas Instruments, Dallas, TX, USA).

Secondly, the developed 64-channel IC was mated at PCB-level with an off-the-shelf 64-channel analogue-to-digital converter (ADC) specialized for medical imaging applications (DDC264, Texas Instruments) as shown in Figure 1. As of writing, this particular family of specialized ADCs exhibits one of the lowest area per channel in the market, a fact which has been exploited in this work to reduce the size of our final two-chip solution as much as possible. When mated together, the custom and off-the-shelf ICs form a small (30 × 34 mm), 64-channel, biosignal acquisition PCB module.

The paper is organized in the following manner: The design methodologies for both the custom CMOS chip and the embedded-level module are detailed in Section 2. The electronic characterization and biological results are provided in Section 3. Discussion and comparison with relevant commercial/academic works are given in Section 4. A summary of the key highlights is provided in Section 5.

## 2. Materials and Methods

### 2.1. Custom ASIC AFE

#### 2.1.1. AC-Coupling Input-Stage

**Figure 2:**
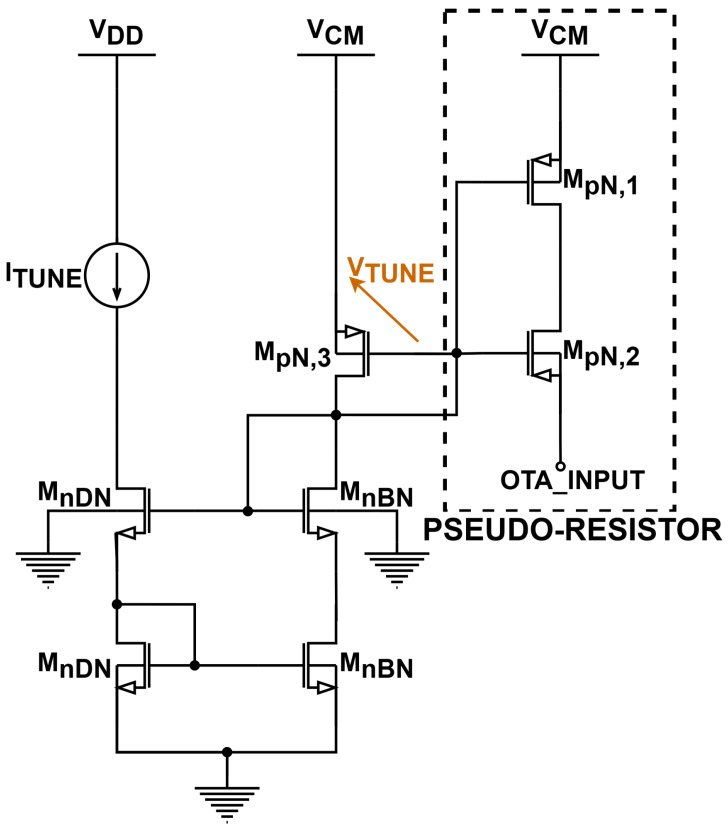
Proposed pseudo-resistor design. Modified from [25]. The tuning voltage $V_{\mathrm{TUNE}}$ is derived by means of the tuning current $I_{\mathrm{TUNE}}$ and sets the resistance value of the pseudo-resistor.

Low frequency signals, below 10 Hz, can sometimes have amplitudes of several orders of magnitude that of sEMG signals. The origin of such interference signals is predominantly related to disturbances in the skin-electrode interface [30]. As such, the sEMG signals should ideally be AC-coupled before amplification to reduce the chance of saturation in the amplification stages. AC-coupling at frequencies between 0.1–10 Hz typically entails RC constants ranging approximately from 2 down to 0.02 s. Due to the limited size of the on-chip capacitors in multi-channel ICs (typically 10–100 pF per channel) resistors in the ranges of 1–100 GΩ are necessary to realize such constants. Transistors in the off-region of operation (also known as pseudo-resistors) can be employed to realize these high resistance elements on-chip [16,31,32,33]. The AC-coupling approach applied in this work makes use of transistors for both the resistive and capacitive elements required to form an AC-coupling/High-pass filter, as has been described in full here [25]. The pseudo-resistor design previously reported was updated slightly to reduce variability across devices (see Figure 2). The tuning voltage source is now tied directly to the common-mode voltage instead of to the drains of both transistors ($M_{pN,3}$ in Figure 2), providing a lower-impedance path for the tuning current to flow. The tuning current mirror ($M_{nDN}//M_{nBN}$ in Figure 2) was also altered from a simple two transistor to a Wilson current mirror device. This alteration was performed to increase the output impedance of the mirror relative to the voltage source transistor $M_{pN,3}$. A Wilson current mirror was chosen over a cascode mirror [34] based on thermal simulations which indicated a marginally lower output current variability across temperature (from 0 to 70 °C).

#### 2.1.2. Operational Transconductance Amplifier

**Figure 3:**
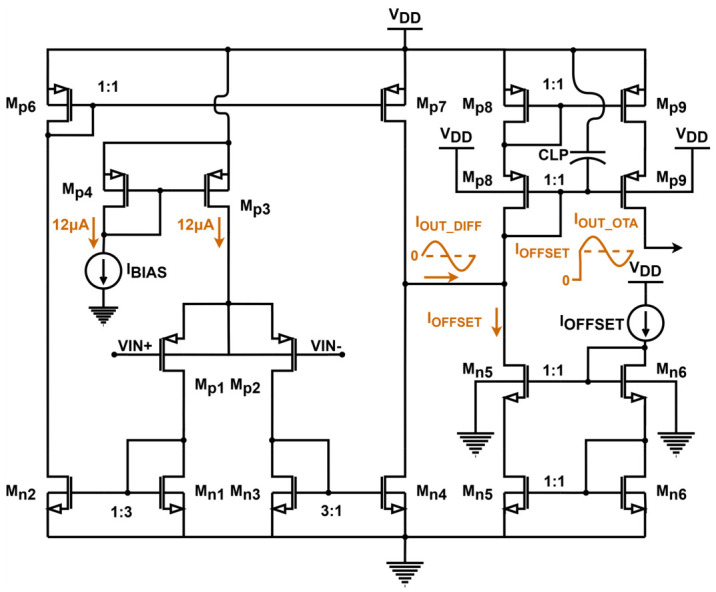
Proposed OTA design. CLP is implemented using a pMOS MOS-CAP device (see Table 1). The current $I_{\mathrm{OFFSET}}$ is summed to the differential output of the OTA $I_{\mathrm{OUT}\_\mathrm{DIFF}}$ to shift the DC point of the final output $I_{\mathrm{OUT}\_\mathrm{OTA}}$ to the middle of the DDC264’s dynamic range, as the DDC264 can only act as a current sink.

A single current-mirror based Operational Transconductance Amplifier (OTA) was used as the amplification unit per channel; as shown in Figure 3. This approach was selected over more complex designs to reduce the number of transistors used and thus enable the utilization of large transistor devices in order to reduce the effect of random process errors and consequently improve performance consistency across channels. The OTA also performs a voltage-to-current conversion on the EMG signal, as required to transform the EMG into a current waveform that can be sampled by the selected specialized ADC converter (DDC264). The OTA was operated in open-loop to ensure that the AC-coupling MOSCAPs and pseudo-resistors are always biased at $V_{CM}$ (Figure 1). Operating at open-loop results in a higher sensitivity of the amplifier to OTA input-offset voltage relative to closed-loop operation. Since the AC dynamic range of the sEMG signal is very small (typical <5 mVpeak) and the DC-level would be filtered before amplification by the AC-coupling circuit, it was deemed feasible to design around this input-offset compromise in order to reduce the complexity and size of the AFE by using the MOSCAPs.

The input differential pair transistors ($M_{p1}$//$M_{p2}$ in Figure 3) have been sized generously at 200/4 μm. The maximum possible area was utilized to reduce noise and input offset voltage. Input-offset variability across channels has also been reduced using these larger transistors to a level comparable to the sEMG signal amplitude; minimizing the maximum output dynamic range required in order to prevent clipping/distortion. PMOS type transistors were used for the differential pair as they exhibit lower flicker noise relative to NMOS type due to the lower mobility of holes vs. electrons. Given the maximum 30 μS transconductance value selected, a 10 mVpp input range specification for the AFE (maximum sEMG signal), and 600 nApp input range of the DDC264 chip in the proposed configuration, the OTA input offset voltage had to be maintained below ±5 mV for all channels to ensure that no channel outputs would fall outside the range of the converter. To further reduce the contribution of input offset from small process variations in the differential pair transistors, the NMOS current mirror loads were implemented at a ratio of input to output of 3:1. The output of the current-mirror OTA was summed to $I_{OFFSET}$ by using a cascode current-mirror ($M_{n5}$–$M_{n6}$ in Figure 3). $I_{OFFSET}$ effectively shifts the DC point of $I_{OUT}$ to the middle of the DDC264’s dynamic range, as the DDC264 can only act as a current sink. A 54.1 pF MOS-based capacitor (CLP in Figure 3; also note the size of $M_{pCLP}$ in Table 1) implemented using a PMOS device was added at the output node of the OTA to limit the bandwidth of the amplifiers below 10 KHz. All OTA transistors were operated in weak-inversion to reduce power consumption. The dimensions of all transistors shown in Figure 1, Figure 2 and Figure 3 are given in Table 1.

#### 2.1.3. Biasing and Power Supply

**Figure 4:**
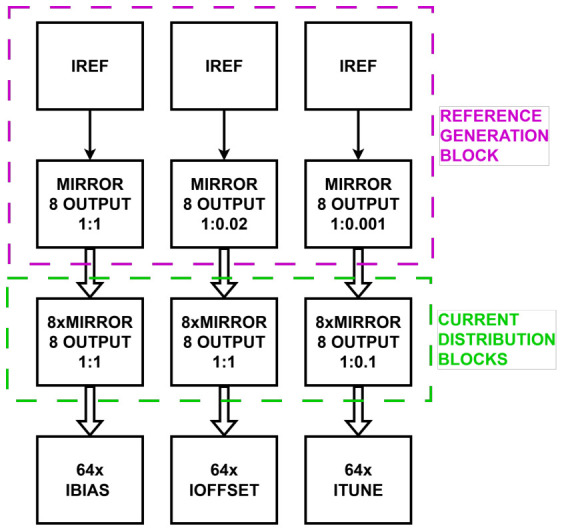
Biasing current generation and distribution approach. Bus-style arrows indicate copying of the same current to multiple outputs. See Figure 5 for the topological realization of the biasing blocks.

The three biasing currents required for this circuit to operate namely, $I_{BIAS}$ (OTA bias), $I_{OFFSET}$ (output offset shift) and $I_{TUNE}$ (pseudo-resistor tuning) were realized from three independent off-chip reference currents to enable individual tuning externally for testing. The reference currents could be generated either by dedicated off-chip current sources or by dropping the supply voltage across off-chip resistors. Reference current magnitudes in the order of 10–100 μA were chosen so that the off-chip resistor value required was large enough to reduce the chip-to-chip variability caused by parasitic in-series resistances. At the same time the resistor value was upper bounded to avoid introducing excessive thermal noise relative to the noise specification of the power supply. Where needed, the reference currents were divided down to the required current values using several stages of on-chip current-mirrors placed in series, as illustrated in Figure 4. For $I_{BIAS}$ and $I_{OFFSET}$ the W/L aspect ratio of the transistors in all current-mirrors were sized so that the transistors operated in strong inversion. For $I_{TUNE}$ the last four stages of current-mirror transistors were sized so that the transistors operated in the sub-threshold region. This was done because the final currents were in the pA range. The proposed design required only one supply rail ($V_{DD}$) at 3 V and consumed approximately 2.3 mA in total. A low-impedance node at mid-level of the supply ($V_{CM}$) was also necessary to bias the pseudo-resistors, with this node drawing approximately 360 pA. Both voltages were supplied using an off-chip commercially-available reference voltage IC (REF1930, Texas Instruments, Dallas, TX, USA [35]).

#### 2.1.4. Layout

**Figure 5:**
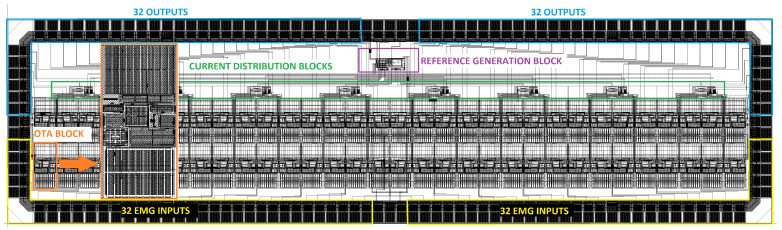
Layout-view of proposed ASIC in 350 nm CMOS technology. Die dimensions (including pads) are 6.9 × 1.8 mm. Each OTA block occupies approximately 200 × 400 μm. Note that approximately 60% of the OTA area is taken-up by MOS-capacitors. The total capacitor value per OTA realized by means of MOS-capacitors amounts to a total of 13.2 pF + 13.2 pF + 54.1 pF = 80.5 pF (the two AC-coupling capacitors and the CLP capacitor).

**Figure 6:**
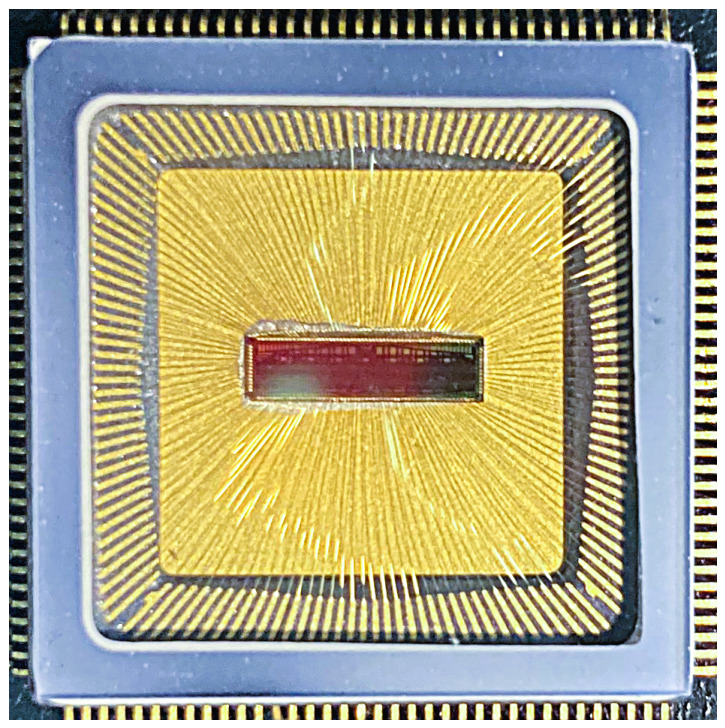
Die in 144-pin TQFP package.

The realized layout is shown in Figure 5. The 64 OTA blocks were arranged in a grid formation of 2 × 32 as a compromise between I/O routing simplicity and minimum chip die aspect ratio (based on IC dicing requirements). The total capacitor value per OTA realized by means of MOS-capacitors amounts to a total of 13.2 pF + 13.2 pF + 54.1 pF = 80.5 pF (the two AC-coupling capacitors and the CLP capacitor). Hence, it is worth noting that the total capacitor area of the ASIC realized by means of MOS-capacitors and corresponding to the 64-channels amounts to 5.152 nF. The reference generation block, containing the $I_{REF}$ generators and first “tree branch” of current mirrors (Figure 4) was placed in the center of the IC. The current distribution blocks, containing the second “tree branch” current-mirrors (Figure 4), were placed closer to individual OTA blocks. Signals flowed along the vertical axis whereas power and biasing currents were distributed along the horizontal to minimize coupling area between signal/power traces. The AFE was packaged in a 144-pin thin-quad-flat-package (TQFP) as illustrated in Figure 6.

### 2.2. PCB-Level Design

**Figure 7:**
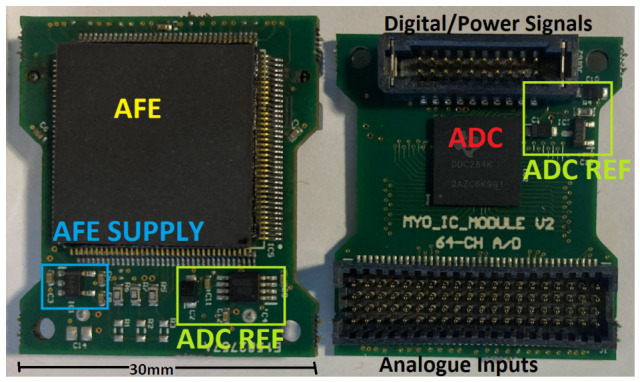
Top and bottom views of realized PCB headstage.

A PCB module was implemented around the proposed IC encapsulating all external analogue circuitry required for data acquisition in a 30 × 34 mm form factor. The module contains our custom AFE IC, an off-the-shelf, 20-bit, current-input ADC (DDC264, Texas Instruments), voltage reference ICs for both the AFE and ADC as well as all associated passives. The realized PCB is shown in Figure 7.

### 2.3. AFE/DDC Interface

The input dynamic range of the system is influenced by the configurations of both DDC264 and the custom AFE IC. The sampling rate and input capacitance of DDC264 are tuneable and directly affect its input dynamic range. Higher sampling rates, resulting in shorter integration times, or larger input capacitance enable measurement of larger current amplitudes. This tuneability can be utilized to manipulate the overall input dynamic range of the combined system. However, when adjusting the input range of the DDC264, the output DC level of the AFE also needs to be tuned via $I_{OFFSET}$ to match the middle of the DDC264’s range. Sampling rate also affects the maximum bandwidth by altering the DDC264’s frequency response. Higher sampling rates result in larger maximum bandwidth at a compromise of increased power consumption. A sampling rate of 4 KHz and maximum input range of 150 pC were selected; resulting in an input range of 600 nApp for the DDC264. Given a maximum expected value of AFE transconductance of 30 μS the resulting maximum input dynamic range of the system would be 20 mVpp. The practical usable AC input dynamic range will be lower when the OTA input-offset voltage is also considered; as it will cause the output DC point of individual channels to shift away from the mid-point of the DDC’s range set by $I_{OFFSET}$ (see Section 2.1.2). Thus, the DDC configuration selected exhibits twice the minimum range needed to capture the desired AC output signal from the AFE to also take into account the variability in input-offset and transconductance across channels within each die ($I_{BIAS}$ and $I_{OFFSET}$ are only externally tuneable for the whole amplifier array). Overall system SNR is unaffected by this design choice due to the very high resolution of the DDC264.

### 2.4. Digital Back-End, Firmware and Software

Programming and data acquisition from the DDC264 has been performed using a Field-Programmable-Gate-Array (FPGA) in this specific case (ICE40-HX8K, Lattice Semi-conductor, Portland, OR, USA). The data were transmitted from the FPGA to a PC using an SPI/USB bridge circuit (FT2232H, Future Technology Devices International, Glasgow, UK).

### 2.5. Electronic Characterization Protocols

The three reference currents for $I_{BIAS}$, $I_{OFFSET}$, $I_{TUNE}$ were set at 12, 12, and 24 μA, respectively, and were all generated off-chip using three precision current sources (Keithley, 6220, Tektronix, Inc., Beaverton, OR, USA). Differential gain against frequency and Total-Harmonic-Distortion (THD) were acquired by applying differential sinusoidal signals at the inputs of the AFE using a signal generator (Keysight, 33500B, Tektronix, Inc., Beaverton, OR, USA) and recording the output of the DDC264. The common-mode gain was measured by applying the same sinusoidal signal to both OTA inputs and the CMRR was calculated based on the ratio of differential gain to common-mode gain. THD and CMRR were evaluated at 60 Hz. The AFE’s input-referred-noise (IRN) was measured by shorting all AFE inputs to ground and recording the output of the DDC264. The value of the output offset current at $I_{OUT,OTA}$ (Figure 3) was determined by taking the average value in a 10 s interval of IRN recording. Crosstalk between adjacent (on the die) channels was evaluated by applying a differential sinusoidal signal of magnitude 10 mVp-p (the largest AC magnitude possible) and of a certain frequency to the positive input of one channel ($V_{SENS+}$ in Figure 1) and pulling down (to system ground) the positive input of an adjacent channel using a 100 KΩ resistor. This resistor value was selected to model the typical impedance of gelled surface electrodes [3]. The negative input/unipolar reference in both channels was tied to the system ground ($V_{SENS-}$ in Figure 1). The outputs of both channels were subsequently recorded and the ratio of the output signal magnitude of the active channel over the signal present at the adjacent channel at the same applied frequency was used to calculate the crosstalk value.

## 3. Results

### 3.1. Electronic Characterization

**Figure 8:**
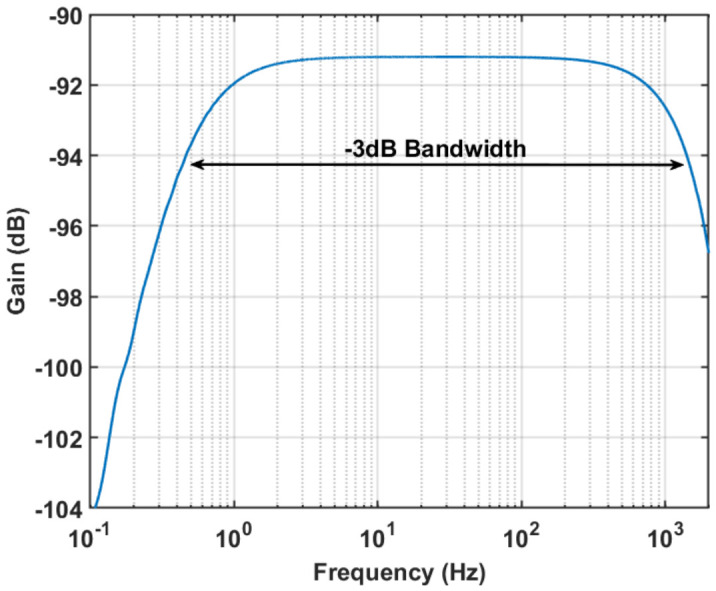
Measured transconductance against frequency averaged across 64-channels from one die. The nominal −3 dB cut-off frequencies for the high-pass and low-pass filters on this die were 0.44 Hz and 1460 Hz, respectively.

The measured transconductance against frequency (bode magnitude plot) averaged across 64 channels from one die is shown in Figure 8. The average measured −3 dB bandwidth for this die was 0.44–1460 Hz.

**Figure 9:**
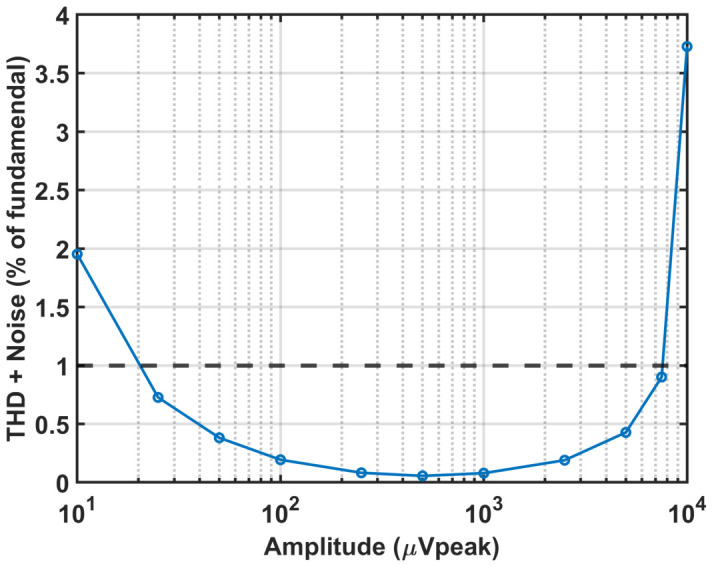
Total-Harmonic-Distortion (THD) and noise across input amplitude. Measured at 60 Hz, average across 64-channels from one die.

**Figure 10:**
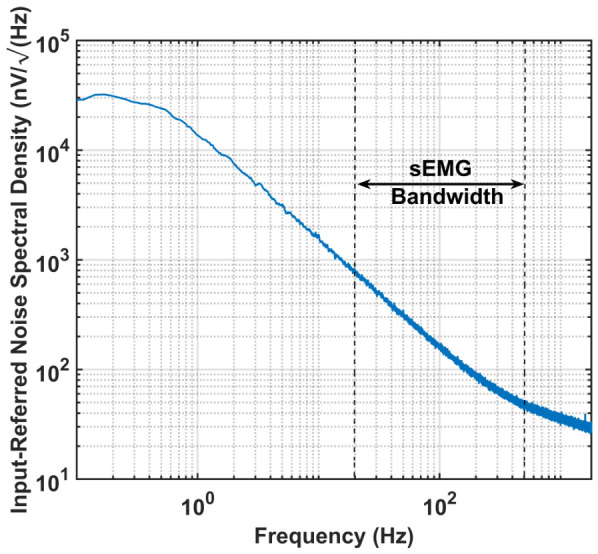
Input-referred-noise spectral density. Averaged across 64-channels from one die. The average IRN integrated within a bandwidth of 20–500 Hz for this die was 3.50 μV_RMS_.

THD against input amplitude averaged across 64 channels from one die is given in Figure 9. The measured output remains below 1% THD for input amplitudes ranging between 20 μVpeak, below which is limited by IRN, and 7.5 mVpeak, above which is limited by the maximum dynamic range of the combination of OTA and DDC264 at the selected configuration (see Section 2.3). Overall the measured linearity results confirm the viability of the adoption of MOS-capacitors as AC-coupling elements in a multi-channel context for hundredths of channels across multiple dies; which to the best of our knowledge is a first. Measurements also confirmed that the device is able to accommodate differential offset voltages of more than ±150 mV for an input signal level of 10 mVp-p; for an input signal level of 5 mVp-p the device is able to accommodate differential offset voltages of ±250 mV [25]. The measured IRN spectral density averaged across 64 channels from one die is illustrated in Figure 10.

**Figure 11:**
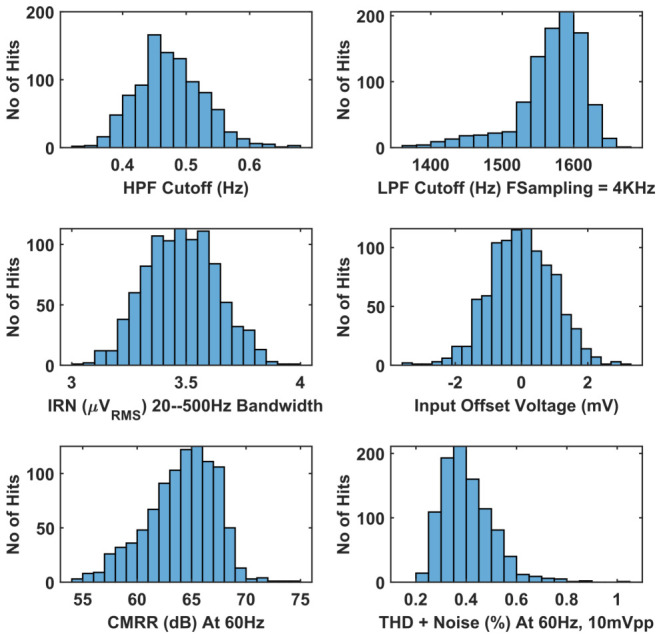
Distribution of Bandwidth, IRN, Input offset, CMRR and THD measured across 960 channels//15 dies. Performance variability across 960 channels indicates that the AFE design approach utilized, using MOS-based capacitors and pseudo-resistors, is a viable approach for multi-channel systems. No significant differences were observed considering inter-chip and intra-chip values.

The distributions of −3 dB bandwidth, IRN (integrated between 20–500 Hz), input offset voltage, CMRR and THD across 960 channels from 15 dies are illustrated in Figure 11.

**Figure 12:**
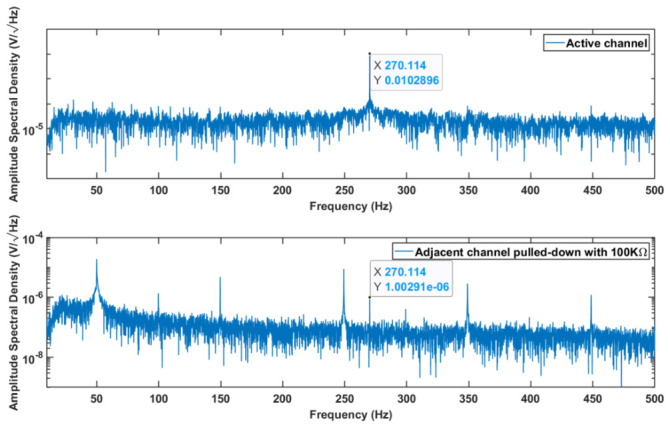
Amplitude spectral density of the recorded signals from both the active and adjacent channels during the crosstalk evaluation at 270 Hz. The raw acquired signals were post-processed using a digital Butterworth band-pass filter of 4^th^ order and pass-band frequency of 20–500 Hz.

Crosstalk was undetectable for frequencies below 130 Hz as the measured signal in the adjacent channel fell under the noise floor of the system. Calculated crosstalk values for frequencies 170, 270, 330, 370, and 470 Hz were 84.1, 80.2, 80.0, 78.3, and 75.3 dB, respectively. The spectra of the measured signals for both the active and adjacent channels which were used to calculate the above values are shown in Figure 12 for an applied frequency of 270 Hz. Table 2 summarizes the properties and performance of our headstage device.

### 3.2. Biological Measurements

**Figure 13:**
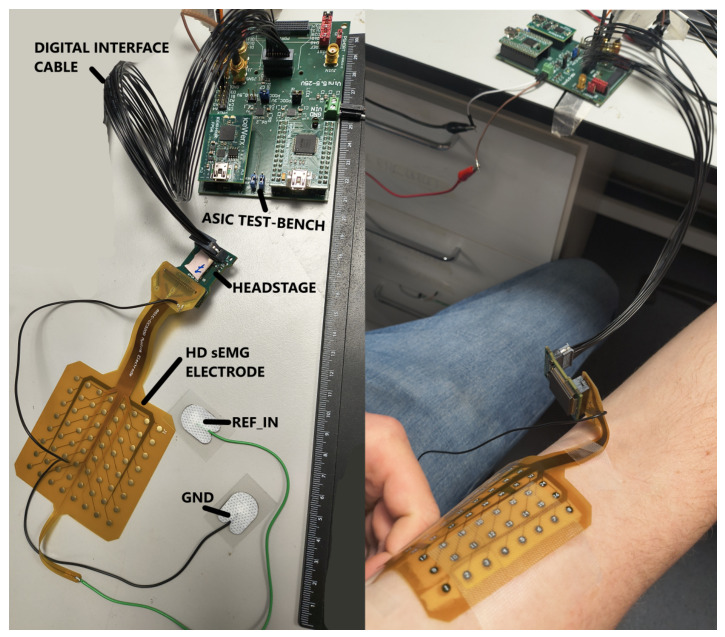
Experimental setup for sEMG acquisition. A custom HD sEMG grid was fabricated and mated to the proposed headstage. The grid electrodes were gelled using the method discussed in [3] and attached to the forearm. The ASIC test-bench contained all digital-back-end circuitry (Section 2.4).

**Figure 14:**
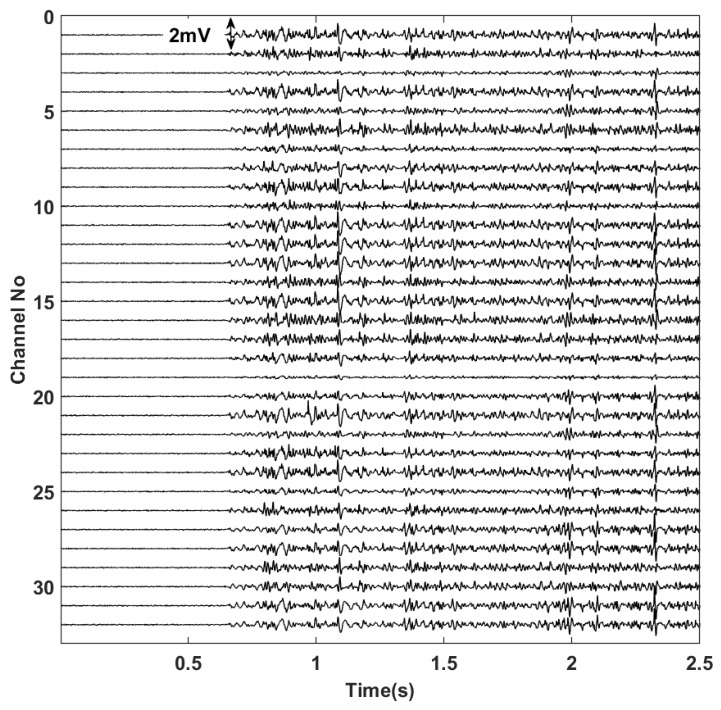
Short 2.5 s snippet of sEMG signals at the onset of muscle contraction acquired from the custom HD sEMG grid shown in Figure 13. The signals were digitally filtered with a 4^th^ order band-pass filter of bandwidth 20–500 Hz and a 2^nd^ order notch filter centered at 50 Hz with a bandwidth of 2 Hz in order to attenuate out-of-band signals and mains interference respectively. A total of 32 out of the 64 recorded channels are displayed here to improve the plotted signal legibility.

Surface EMG signals were acquired from the forearm with the developed system using a custom 64-channel HD sEMG grid. The negative inputs for all AFE channels were connected to a reference electrode placed on the elbow bone. A ground electrode was also placed on the elbow. Pre-gelled electrodes (Spes Medica, DENIS01526, Spes Medica S.p.A., Genova, Italy) were used for the ground and reference electrodes. The experimental setup and a snippet of recorded signals are shown in Figure 13 and Figure 14, respectively.

## 4. Discussion

### 4.1. Integrated Analogue Front-End

A custom designed 64-channel AFE, occupying less than 0.2 mm^2^ per channel has been fabricated in 350 nm CMOS process and 960 individual channels have been characterized from 15 dies in 2 tape-outs. The measured performance characteristics of the proposed AFE are listed along with the corresponding values reported by comparable implementations in Table 3. When considering the ICs which are proposed for the same application [36,37,38,39,40], the proposed approach occupies one of the lowest area/channel at 0.19 mm^2^ despite using an older technology which is characterized by a larger feature size; enabling a more area-effective scale-up to higher numbers of HD sEMG channels. Work [37] has a lower area per channel at 0.12 mm^2^ but utilizes a 65nm technology which would be much costlier to scale-up. Moreover, the actual area per channel occupied by the circuitry is about 30% lower (0.133 mm^2^) as some additional “empty” die area was added to improve IC dicing yield due to the rectangular die aspect ratio chosen. This area reduction has been primarily thanks to the use of MOS capacitors to implement both the on-chip AC-coupling filters, as was also done in [29], and the on-chip low-pass filters, as was also performed by [40,41]. The use of a simplified open-loop amplifier topology [29,41] further contributes to the area reduction. Multi-channel ICs built with smaller feature size technologies and targeting neural/implantable applications exist with significantly lower area per channel [17], however such areas are typically achieved by using smaller value AC coupling capacitors. Such an approach makes the AC-coupled node more susceptible to parasitic capacitances of the pseudo-resistor and input-pair.

Power consumption per channel is slightly greater than in many previous designs (95 μW from a 3 V supply); however, it is acceptable for non-implantable applications (particularly when compared to general-purpose off-the-shelf amplifiers). For example, a 1024-channel system utilizing this AFE would consume approximately 100 mW. The CMRR of the proposed device is lower than other ICs targeting the same application at a typical value of 65 dB. This is a trade-off of the simplified topology used to reduce per-channel area. The resulting compact front-end implementation enables placement of the whole acquisition system in very close proximity to the electrodes, as shown in Figure 13, thus reducing the amplitude of common-mode electromagnetic interference present on the recordings and relaxing the practical CMRR requirement as discussed in Section 1. IRN and/or CMRR can be improved using chopper stabilization as illustrated by [38,41]. However, for this specific application, as the majority of the bandwidth of interest is above 20 Hz, flicker noise is typically not as critical as in ECG/EEG applications. A simpler approach to achieving very low IRN using capacitively-coupled operational transconductance amplifiers (CC-OTAs) is to shift the high-pass cut-off well below 0.1 Hz and effectively utilize the AC coupling capacitor to de-couple the pseudo-resistor Brown noise from the amplifier inputs [16,42]. This can work; however, practical considerations must also be taken into account based on the desired implementation; such as the settling time of the amplifier and variability of the high-pass cut-off across channels. Alternatively, increasing the input capacitance and effectively reducing the pseudo-resistor impedance for the same cut-off can also be utilized to improve IRN performance at the compromise of IC area (by reducing the Brown noise contribution of the pseudo-resistors). The analogue high-pass cut-off frequency in this work was set at 0.5 Hz as a compromise between area, noise and off-band attenuation levels for the sEMG application. A minimum 10 mVp-p AFE input dynamic range was designed and verified by the THD measurements to enable acquisition of the full sEMG amplitude of interest. During our biological testing any residual motion artifacts fell within the AC dynamic range of the system and were subsequently digitally filtered. In setups where the motion artifact amplitude would be large enough to risk AFE clipping, the system’s dynamic range could be increased by reducing the OTA gain (see Section 2.1.3) or by increasing the DDC’s sampling rate (see Section 2.3). After taking into account the bandwidth of IRN measurement, the proposed AFE IRN is on-par with works that utilize more complex/costly designs [36,37,38,39,40].

### 4.2. Prototype Headstage

A prototype headstage optimized for HD sEMG acquisition has been realized using a custom 64-channel AFE and an off-the-shelf multi-channel ADC specialized for medical imaging applications (two-chip solution). Key performance specifications of the developed headstage are compared with those of relevant devices, both commercially-available as well as reported research, in Table 4. The proposed device exhibits comparable IRN, power dissipation and sufficient sampling rate for the sEMG application relative to solutions implemented with off-the-shelf amplifiers in DC-coupled configuration [43]. The Intan single-chip ADC and AFE solution consumes less power and also has a slightly lower IRN floor (based on our estimates given the values provided on the device’s datasheet) with the proposed solution exhibiting higher sampling resolution. The IMEC NeuroPixels headstage performs slightly better in terms of area per channel and power consumption but exhibits a slightly higher noise floor and lower resolution. The majority of area and mass of the proposed two-chip solution results from the prototype TQFP IC package used. A custom Ball-Grid-Array package could be utilized to significantly reduce the PCB area and mass. Further PCBA area reductions can be achieved by incorporating all the supporting analogue circuitry (biasing current sources and common-mode reference voltage) into the 64-channel ASIC. Given the 6.5 × 0.95 mm of core area utilized by the AFEs, a mixed-signal chip realization, similar to the Intan RHD2164, would be feasible based on the developed IC at a die footprint of approximately half the size (Intan RHD2164 die is approximately 7 × 4 mm), and by extension lower IC cost. This is partly the result of the low AFE area achieved and partly due to the removal of additional features, such as on-chip filter tuning and temperature sensors, which are not required for sEMG applications. The noise floor of the proposed AFE exceeds the quantization resolution of the selected off-chip ADC module. Given dynamic range and noise considerations an ADC with noise-free resolution of 12.2-bits would be sufficient to capture the full range supported by the AFE. However, when considering other options on the market, the selected ADC delivers one of the lowest PCB area per channel without significant compromise in quantization performance. The performance/area optimizations performed by the manufacturer to make it suitable for X-ray detection application translate well to the problem of HD sEMG acquisition; where channel footprint becomes more and more critical as the number of channels increases. Moreover, using this off-the-shelf back-end, scale-up becomes enviably straightforward as different versions of the same IC are available with up to 256-channels per ADC; an enticing feature which is rarely shared by other chipset families while preserving minimal PCB area per channel. In implementations where data rate needs to be reduced, such as when hundreds of channels are recorded simultaneously or when the data are to be streamed wirelessly, this ADC can be operated in 16-bit resolution mode. Furthermore, it is worth stressing that the low-volume prototype fabrication cost for the fifteen headstages presented in this work was approximately 55% of the off-the-shelf price for fifteen 64-channel Intan modules (Table 4) at the time of writing and approximately the same cost as fifteen 64-channel implementations utilizing generic off-the-shelf components and discrete passives; which would take-up almost double the board area to the solution proposed. This approximate estimation suggests that potential volume production of the proposed headstage could result in very competitive cost (and price) per unit for the given board area. Lastly, when compared to state-of-the-art commercially-available HD sEMG probes with identical channel count, the proposed headstage offers comparable IRN performance to the OTB Muovi+ while occupying under 25% of the system’s volume and slightly inferior IRN performance than the TMSI SPIRE+ but whilst occupying under 10% its volume. As the electrode-skin noise levels tend to exceed several μV_RMS_ unless in very well controlled lab conditions [3] the lower noise level of the TMSI device is, in our opinion, unlikely to facilitate significantly improved SNR in typical wearable setups. Given that the majority of area inside such systems is dictated by the AFE size it can be argued that a 64-channel wireless probe built using the proposed headstage would occupy a significantly smaller footprint than both the OTB and TMSI probes and thus provide higher freedom of movement when attached to the patient.

## 5. Conclusions

A miniature headstage circuit optimized for recording HD sEMG has been designed, implemented and characterized (960 channels tested). The circuit combines a custom ASIC front-end, developed in 350 nm CMOS technology, with an off-the-shelf ADC which is typically used in medical imaging systems. All filter sub-circuits in the custom ASIC have been realized using MOS devices only in order to reduce cost (and area) as much as possible. To the best of our knowledge this is the first realization and demonstration of a MOS only AFE for sEMG, the first MOS only AFE realization that has been assessed by measurements against so many channels, as well as the first use of an X-ray detection ADC to measure surface EMG signals. Both demonstrated solutions can be utilized autonomously or in-tandem to reduce the cost for high-density surface biosignal acquisition. 

## Figures and Tables

**Table 1:** Transistor dimensions for the sub-circuits shown in Figure 1, Figure 2 and Figure 3.

Transistor	Width (μm)	Length (μm)
$M_{pCIN}$	2970	1
$M_{pCLP}$	6000	2
$M_{pN,3}$	30	6
$M_{pN,1–2}$	30	6
$M_{nBN}$	3	15
$M_{nDN}$	15	15
$M_{n1,3}$	18	54
$M_{n2,4}$	6	54
$M_{n5,6}$	7.5	10
$M_{p1,2}$	200	4
$M_{p3,4}$	30	5
$M_{p6,7}$	7	32
$M_{p8,9}$	10	30

**Table 2:** Summary of typical performance characteristics of the proposed MOS-only two-chip 64-channel headstage. See Figure 11 for performance variability across 960 channels.

Parameter	Value	Unit	Conditions
No of EMG Channels	64	-	-
Configuration	Unipolar	-	All against Reference electrode
Input AC Range	±5	mV	Input DC offset within linear range
Input DC Suppression	±150	mV	Differential vs. Reference
Input MOS Capacitance (Working electrode)	13.2	pF	Input DC offset within linear range
Input MOS Capacitance (Reference electrode)	844.8	pF	Input DC offset within linear range
Transconductance	28	μS	-
Maximum Bandwidth	0.45–1600	Hz	-
THD	<0.9	%	60 Hz, ±5 mV AC signal
CMRR	65	dB	60 Hz
IRN	3.5	μV_RMS_	20–500 Hz Bandwidth
Crosstalk (between two adjacent channels on the die)	80.2	dB	270 Hz, 100 KΩ pull-down resistor on input of adjacent chnannel
Resolution	20	bit	-
Sampling Rate	3500–6000	Hz	Firmware Tuneable
ASIC CMOS Technology	0.35	μm	-
ASIC Supply Voltage	3	V	-
ASIC Power Consumption	7	mW	ASIC AVDD = 3 V
ASIC Area	12.42	mm^2^	-
Headstage Power Consumption	320	mW	4 KHz Sampling Rate
Headstage Dimensions	30 × 34 × 10	mm	-
Headstage Mass	8.6	g	-

**Table 3:** Comparison of proposed AFE with relevant integrated-level multi-channel front-ends. Mean value measured across 960 AFEs is given for this work. * Area including on-chip ADC. ** IRN measured at full Bandwidth stated. *** Power includes on-chip ADC.

Work	Target	Input-Stage Architecture	MOS-CAPs	TECH (nm)	No CH per IC	No CH Tested	Area per CH (mm^2^)	Power per CH (μW)	IRN (μV_RMS_)	Band-Width (Hz)	CMRR (dB)	THD (%)
This Work	sEMG	OL CC OTA	YES	350	64	960	0.19	95 (3 V)	3.48 (20–500 Hz)	0.47–1.6k	64.1	0.41 (5 mVpk)
[36]	sEMG	CL CC OTA	NO	180	16	16	0.40	40 (1.5 V)	7.91 (20–500 Hz)	10–500k	-	<0.1 (5 mVpk)
[29]	Neural	OL CC OTA	YES	180	64	64	0.18	220	6 **	20–6.5k	-	-
[37]	sEXG	CL CC INA	NO	65	7	7	0.12	2.6 (1.2 V)	1.24 **	0.5–250	91	0.14 (0.5 mVpk)
[38]	sEXG	CL CC OTA	NO	180	8	-	0.34	56 (1.8 V)	5.1 **	0.5–10k	105	-
[41]	Neural	OL CC OTA	YES	130	96	276	0.26 *	35 (1.2 V)	14 (1–100 Hz)	1–10k	-	-
[17]	Neural	CL CC OTA	NO	180	128	128	0.06	3 (1 V) ***	3.3 **	0.5–12.7k	>60	0.02 (1.5 mVpk)
[39]	sEXG	CL CC IA	NO	180	4	4	0.75 *	43.8 (1.8 V) ***	3.52 **	1–150	76	-
[40]	sEXG	CL CC LNA	YES	180	4	4	0.37 *	0.37 (1 V) ***	2.9 **	0.2–128	81.6	2.75 (2 mVpk)

**Table 4:** Comparison of proposed headstage with relevant embedded-level multi-channel acquisition boards as well as two state-of-the-art commercially available HD sEMG probes. Mean value measured across 960 AFEs is given for this work. * Dimensions/mass including battery and case. ** Power including wireless transmission.

Device	No CH	Board Topology	PCB Dimensions (mm)	Mass (g)	Powerper CH (mW)	Sampling Rate (KHz)	Resolution	Dynamic Range (mVpp)	IRN (μV_RMS_)
This work	64	AFE + ADC (two-chip)	30 × 34 × 10	8.6	5	2–6	20-bit	>10	3.5 (20–500 Hz)
[43]	60	AFE Only	29 × 21 × 6	3.0	3.8	2	18-bit	480	5.9 (0–300 Hz)
RHD2164 PCB	64	AFE + ADC (single-chip)	22 × 14 × 5	1.3	<1 (Estimate, [20])	1–30	16-bit	10	<2 (Estimate, 20–500 Hz [20])
OPEN BCI Cyton	8	AFE + ADC (single-chip)	61 × 61× 8	-	4.8 (ADS1299 [44])	0.250	24-bit	5000	1.1 (DC-65 Hz)
Neuro-Pixels 1.0	128	AFE + ADC (single-chip)	24 × 22 × 5	3.3	<0.1	2.5	10-bit	10	9.2 (0.5–500 Hz)
OTB Muovi+ probe	64	N/A	50 × 45 × 20 *	42 *	9.4 **	2	16-bit	1000	<6 (DC-500)
TMSI SPIRE+ probe	64	N/A	91 × 65 × 21 *	120 *	N/A	2	24-bit	300	<1.8 (DC-500)

## Data Availability

Data available on request.

## References

[1] Merletti R., Cerone G. (2020). Tutorial. Surface EMG detection, conditioning and pre-processing: Best practices. J. Electromyogr. Kinesiol..

[2] Clancy E., Morin E., Merletti R. (2002). Sampling, noise-reduction and amplitude estimation issues in surface electromyography. J. Electromyogr. Kinesiol..

[3] Piervirgili G., Petracca F., Merletti R. (2014). A new method to assess skin treatments for lowering the impedance and noise of individual gelled Ag-AgCl electrodes. Physiol. Meas..

[4] Zwarts M.J., Stegeman D.F. (2003). Multichannel surface EMG: Basic aspects and clinical utility. Muscle Nerve.

[5] Farina D., Holobar A. (2016). Characterization of Human Motor Units from Surface EMG Decomposition. Proc. IEEE.

[6] Drost G., Stegeman D.F., van Engelen B.G., Zwarts M.J. (2006). Clinical applications of high-density surface EMG: A systematic review. J. Electromyogr. Kinesiol..

[7] Kapelner T., Jiang N., Holobar A., Vujaklija I., Roche A.D., Farina D., Aszmann O.C. (2016). Motor Unit Characteristics after Targeted Muscle Reinnervation. PLoS ONE.

[8] Geng W., Du Y., Jin W., Wei W., Hu Y., Li J. (2016). Gesture recognition by instantaneous surface EMG images. Sci. Rep..

[9] Tam S., Boukadoum M., Campeau-Lecours A., Gosselin B. (2020). A Fully Embedded Adaptive Real-Time Hand Gesture Classifier Leveraging HD-sEMG and Deep Learning. IEEE Trans. Biomed. Circuits Syst..

[10] Kapelner T., Sartori M., Negro F., Farina D. (2020). Neuro-Musculoskeletal Mapping for Man-Machine Interfacing. Sci. Rep..

[11] Barsakcioglu D.Y., Farina D. (2018). A real-time surface EMG decomposition system for non-invasive human-machine interfaces. Proceedings of the 2018 IEEE Biomedical Circuits and Systems Conference (BioCAS).

[12] Jiang X., Xu K., Liu X., Dai C., Clifton D.A., Clancy E.A., Akay M., Chen W. (2021). Neuromuscular Password-Based User Authentication. IEEE Trans. Ind. Inform..

[13] Lu L., Mao J., Wang W., Ding G., Zhang Z. (2020). A Study of Personal Recognition Method Based on EMG Signal. IEEE Trans. Biomed. Circuits Syst..

[14] Muller R., Gambini S., Rabaey J.M. (2012). A 0.013 mm^2^, 5 μW, DC-Coupled Neural Signal Acquisition IC with 0.5 V Supply. IEEE J. Solid State Circuits.

[15] Papadopoulou A., Hermiz J., Grace C., Denes P. (2024). A Modular 512-Channel Neural Signal Acquisition ASIC for High-Density 4096 Channel Electrophysiology. Sensors.

[16] Harrison R.R., Charles C. (2003). A low-power low-noise CMOS amplifier for neural recording applications. IEEE J. Solid State Circuits.

[17] Park S.Y., Cho J., Na K., Yoon E. (2018). Modular 128-Channel Δ-ΔΣ Analog Front-End Architecture Using Spectrum Equalization Scheme for 1024-Channel 3-D Neural Recording Microsystems. IEEE J. Solid State Circuits.

[18] Samiei A., Hashemi H. (2019). A Chopper Stabilized, Current Feedback, Neural Recording Amplifier. IEEE Solid State Circuits Lett..

[19] Yazicioglu R.F., Merken P., Puers R., Van Hoof C. (2008). A 200 μW Eight-Channel EEG Acquisition ASIC for Ambulatory EEG Systems. IEEE J. Solid State Circuits.

[20] Intan Technologies (2012). RHD2000 Series Digital Electrophysiology Interface Chips Datasheet.

[21] Texas Instruments (2010). Low-Power, 8-Channel, 16-Bit Analog Front-End for Biopotential Measurements Datasheet.

[22] Texas Instruments (2006). DDC264 64-Channel, Current-Input Analog-to-Digital Converter Datasheet.

[23] Trumpis M., Insanally M., Zou J., Elsharif A., Ghomashchi A., Sertac Artan N., Froemke R.C., Viventi J. (2017). A low-cost, scalable, current-sensing digital headstage for high channel count *μ*ECoG. J. Neural Eng..

[24] Houssein A., Drakakis E. (2017). MOS-only reduced-order ELIN cochlear channels: Comparative performance evaluation. Int. J. Circuit Theory Appl..

[25] Koutsoftidis S., Belgaid Y., Yang G., Barsakcioglu D.Y., Glaros K.N., Farina D., Drakakis E.M. (2024). A capacitorless, AC-coupled, monolithic input-stage optimized for multi-channel surface EMG acquisition. Proceedings of the 2024 46th Annual International Conference of the IEEE Engineering in Medicine and Biology Society (EMBC).

[26] Ghamati M., Maymandi-Nejad M. A low-noise low-power MOSFET only electrocardiogram amplifier. Proceedings of the 2013 21st Iranian Conference on Electrical Engineering (ICEE).

[27] Kuo K.C., Chen C.T., Liao H.Y. An Area Efficient Analog Front-End for Sensing EEG Signals with MOS Capacitors in 90nm Process. Proceedings of the 2023 International Conference on Consumer Electronics—Taiwan (ICCE-Taiwan).

[28] Chaturvedi V., Amrutur B. (2011). An Area-Efficient Noise-Adaptive Neural Amplifier in 130 nm CMOS Technology. IEEE J. Emerg. Sel. Top. Circuits Syst..

[29] Kmon P., Zoladz M., Grybos P., Szczygiel R. (2009). Design and measurements of 64-channel ASIC for neural signal recording. Proceedings of the 2009 Annual International Conference of the IEEE Engineering in Medicine and Biology Society.

[30] Huigen E., Peper A., Grimbergen C.A. (2002). Investigation into the origin of the noise of surface electrodes. Med. Biol. Eng. Comput..

[31] Djekic D., Ortmanns M., Fantner G., Anders J. A tunable, robust pseudo-resistor with enhanced linearity for scanning ion-conductance microscopy. Proceedings of the 2016 IEEE International Symposium on Circuits and Systems (ISCAS).

[32] Guglielmi E., Toso F., Zanetto F., Sciortino G., Mesri A., Sampietro M., Ferrari G. (2020). High-value tunable pseudo-resistors design. IEEE J. Solid State Circuits.

[33] Trzpil-Jurgielewicz B., Dąbrowski W., Hottowy P. (2021). Analysis and Reduction of Nonlinear Distortion in AC-Coupled CMOS Neural Amplifiers with Tunable Cutoff Frequencies. Sensors.

[34] Carusone T.C., Johns D., Martin K.W.K.W., Carusone T.C., Johns D.A., Martin K.W. (2013). Analog Integrated Circuit Design.

[35] Texas Instruments (2014). REF19xx Low-Drift, Low-Power, Dual-Output, VREF and VREF/2 Voltage References Datasheet.

[36] Huang Y.K., Schrunder A.F., Rusu A., Rodriguez S. (2024). A Frequency-Division Multiplexed 16-Channel AFE for Wearable MC-sEMG Recording. Proceedings of the 2024 31st IEEE International Conference on Electronics, Circuits and Systems (ICECS).

[37] Yin J., Liu X., Zhang Y., Liao X., Liu L. (2025). A Low-Channel-Mismatch ExG AFE Based on Orthogonal Nested-Chopper and Successive-Approximation Input Capacitance Calibration. IEEE Sens. J..

[38] Chen W.M., Yang W.C., Tsai T.Y., Chiueh H., Wu C.Y. (2011). The design of CMOS general-purpose analog front-end circuit with tunable gain and bandwidth for biopotential signal recording systems. Proceedings of the 2011 Annual International Conference of the IEEE Engineering in Medicine and Biology Society.

[39] Kim J., Ouh H., Johnston M.L. (2021). Multi-Channel Biopotential Acquisition System Using Frequency-Division Multiplexing with Cable Motion Artifact Suppression. IEEE Trans. Biomed. Circuits Syst..

[40] Fath P., Pretl H. (2024). A 370-nW Bio-AFE with 2.9-μ Vrms Input Noise in an Octa-Channel System-in-Package for Multimode Bio-Signal Acquisition. IEEE Trans. Very Large Scale Integr. (VLSI) Syst..

[41] Gao H., Walker R.M., Nuyujukian P., Makinwa K.A.A., Shenoy K.V., Murmann B., Meng T.H. (2012). HermesE: A 96-Channel Full Data Rate Direct Neural Interface in 0.13 *μ* m CMOS. IEEE J. Solid State Circuits.

[42] Serrano-Finetti E., Pallas-Areny R. (2014). Noise Reduction in AC-Coupled Amplifiers. IEEE Trans. Instrum. Meas..

[43] Wang J., Trumpis M., Insanally M., Froemke R., Viventi J. (2014). A low-cost, multiplexed electrophysiology system for chronic *μ*ECoG recordings in rodents. Proceedings of the 2014 36th Annual International Conference of the IEEE Engineering in Medicine and Biology Society.

[44] Texas Instruments (2012). ADS1299-x Low-Noise, 4-, 6-, 8-Channel, 24-Bit, Analog-to-Digital Converter for EEG and Biopotential Measurements Datasheet.

